# Pregnane X Receptor Signaling Pathway and Vitamin K: Molecular Mechanisms and Clinical Relevance in Human Health

**DOI:** 10.3390/cells13080681

**Published:** 2024-04-14

**Authors:** Jeff L. Staudinger, Avina Mahroke, Gauri Patel, Cole Dattel, Sahana Reddy

**Affiliations:** Division of Basic Sciences, Farber-McIntire Campus, College of Osteopathic Medicine, Kansas City University, Joplin Campus, 2901 St Johns Blvd, Joplin, MO 64804, USAcole.dattel@kansascity.edu (C.D.); sahana.reddy@kansascity.edu (S.R.)

**Keywords:** PXR activation, Pregnane X Receptor, vitamin K2, gut health, liver health, clinical implications, inflammatory bowel disease (IBD), cancer, colorectal cancer, autophagy, oxidative stress, inflammation, xenobiotic metabolism, bone health, precision medicine, therapeutic interventions

## Abstract

This review explores the likely clinical impact of Pregnane X Receptor (PXR) activation by vitamin K on human health. PXR, initially recognized as a master regulator of xenobiotic metabolism in liver, emerges as a key regulator influencing intestinal homeostasis, inflammation, oxidative stress, and autophagy. The activation of PXR by vitamin K highlights its role as a potent endogenous and local agonist with diverse clinical implications. Recent research suggests that the vitamin K-mediated activation of PXR highlights this vitamin’s potential in addressing pathophysiological conditions by promoting hepatic detoxification, fortifying gut barrier integrity, and controlling pro-inflammatory and apoptotic pathways. PXR activation by vitamin K provides an intricate association with cancer cell survival, particularly in colorectal and liver cancers, to provide new insights into potential novel therapeutic strategies. Understanding the clinical implications of PXR activation by vitamin K bridges molecular mechanisms with health outcomes, further offering personalized therapeutic approaches for complex diseases.

## 1. Introduction

The PXR protein, encoded by the NR1I2 gene, is a vital component of the human gut and liver regulatory system [1,2,3,4]. As a nuclear receptor, PXR plays a central role in the modulation of xenobiotic metabolism and detoxification processes, primarily in liver and intestine. As a ligand-activated transcription factor, PXR is primarily responsible for regulating the expression of genes encoding various drug-metabolizing enzymes and drug-transporter proteins, thus aiding the clearance of xenobiotics from the body [5,6,7,8,9]. However, its significance goes beyond xenobiotic metabolism. PXR serves as a sentinel that integrates endogenous and exogenous signals to facilitate adaptation of the physiological and metabolic functions in these organs in response to environmental cues. Its ligand-mediated activation and subsequent post-translational modification leads to a cascade of downstream events affecting not only drug metabolism but also vitamin D metabolism, bone formation, inflammation, autophagy, oxidative stress, and even cell survival [10,11,12,13,14,15,16,17,18,19,20].

For additional recent updates regarding PXR biology, the reader is referred to several excellent review articles. Specifically, a 2021 review article by Dutta and colleagues highlights an important emerging paradigm in which the gut microbiome is gaining recognition as a significant regulator of host xenobiotic biotransformation and intermediary metabolism, with PXR playing a role in both regulating and being regulated by the gut–liver axis [10]. For more information regarding the biology of post-translational modification of the PXR protein, Rogers and colleagues provide a significant 2021 review of the interface between cell signaling pathways and PXR [21]. Recent research efforts highlight numerous post-translational modifications (PTMs) which significantly influence the biological function of PXR. However, this thrust of research is still in its infancy. This review provides a discussion of how these PTMs likely interface with each other to respond to extracellular cues to appropriately modify PXR activity. A concise 2020 review manuscript regarding the likely potential role for PXR in breast cancer is provided by Creamer et al. [22]. The elevated level of PXR expression in cancerous breast tissue suggests a likely interface between aberrant cell division and xeno-protection in cancer cells. Moreover, PXR itself exerts a positive effect on the cell cycle, thereby predisposing tumor cells to unchecked proliferation. The activation of PXR also likely plays a key role in regulating apoptosis, as well as in acquired resistance to chemotherapeutic agents.

**Figure 1:**
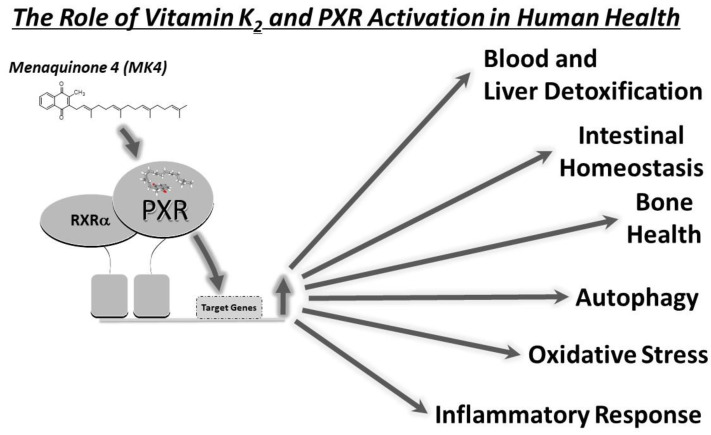
The scheme illustrates a likely key role of vitamin K2 (MK-4) and PXR activation in human health. MK-4-mediated activation of PXR plays a pivotal role in detoxification of blood, liver, bone, and gut. PXR activation accomplishes these biological functions through regulating the expression and activity of genes involved in autophagy, oxidative stress, and the inflammatory response.

The rationale for this review is rooted in the recent expanding knowledge of PXR activation by vitamin K2 and its potential impact on overall human health. Emerging research suggests that PXR activation by vitamin K2 can influence processes critical to gut, liver, and bone health, including the intricate balance of inflammation, oxidative stress, and cancer cell survival in these tissues [23,24,25,26,27,28,29,30,31,32]. In response to the growing interest in the therapeutic potential of vitamin K2, this review aims to bridge the gap between its fundamental molecular mechanisms and likely clinical applications of PXR activation by this compound. It will explore how the interplay between vitamin K2 and PXR holds potential for improving outcomes in conditions such as inflammatory bowel disease, cancer, osteoporosis, and other disorders linked to overall human health (Figure 1).

## 2. Vitamin K and γ-Glutamyl Carboxylase

**Figure 2:**
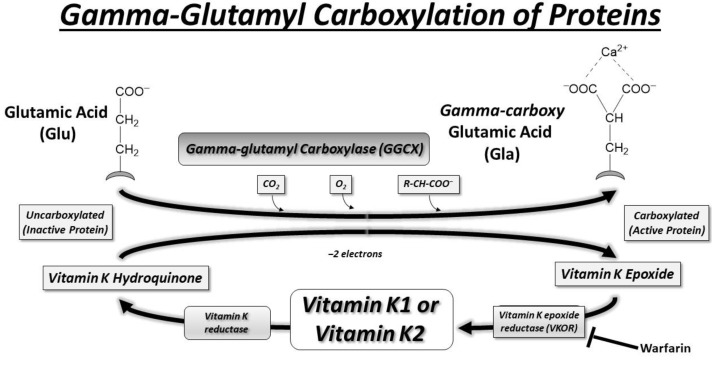
The scheme illustrates the pathway used to accomplish the gamma-glutamyl carboxylation of proteins using vitamin K as a cofactor. Vitamin K plays a crucial role in the synthesis of Gla proteins by serving as a cofactor for the GGCX enzyme, which catalyzes the conversion of glutamic acid residues to gamma-carboxyglutamic acid residues. The GGCX enzyme can utilize both vitamin K1 and vitamin K2 in the gamma-carboxylation process, and the choice of cofactor depends on the specific Gla protein being modified and its location within the body. One significant biological consequence of gamma-glutamyl carboxylation of clotting factors II, VII, IX, and X is the ability to bind the released Calcium (Ca^2+^) ions and form the tenase complex at the site of blood vessel injury.

Danish biochemist Carl Peter Henrik Dam received the Nobel Prize in Physiology or Medicine in 1943 for his groundbreaking discovery of vitamin K, the essential factor in blood coagulation [33]. He shared this prestigious prize with American biochemist Edward A. Doisy (1893–1986), who independently conducted research in St. Louis, Missouri, leading to the identification of the chemical nature of vitamin K [34]. Vitamin K was first recognized to act as a cofactor for gamma-glutamyl carboxylase (GGCX) in the early 1970s [35,36]. The discovery of the role of vitamin K in the carboxylation of certain proteins, including GGCX, was crucial for understanding its importance in blood clotting and bone metabolism [37]. This vitamin K-dependent carboxylation process is essential for the activation of various proteins involved in these physiological functions and is depicted in the scheme in Figure 2. Gamma-glutamyl carboxylation is an enzymatic modification that involves the addition of gamma-carboxyglutamic acid (Gla) residues to specific glutamic acid (Glu) residues within a protein structure, resulting in the formation of a group of proteins termed ‘Gla proteins’.

In the early 20th century, Canadian farmers observed a bleeding disorder in their cattle that occurred after the animals consumed spoiled sweet clover hay. This condition, dubbed “sweet clover disease” or “bleeding disease”, prompted researchers in Canada to investigate its cause during the 1920s and 1930s. Their exploration led to the identification of dicoumarol in the spoiled sweet clover hay as the culprit behind the anticoagulant effect responsible for the bleeding in cattle [38]. Dicoumarol, a coumarin derivative, interfered with vitamin K metabolism, hindering the synthesis of functional clotting factors. In 1948, Wisconsin biochemist Karl Paul Link and his team successfully isolated and synthesized a more potent and specific compound derived from dicoumarol, naming it warfarin [38]. The term “warfarin” is derived from WARF, the Wisconsin Alumni Research Foundation, where much of the initial research occurred [39]. Warfarin was subsequently found to be effective in preventing the formation of blood clots by inhibiting vitamin K-dependent clotting factors.

## 3. Gla Proteins and Human Health

**Figure 3:**
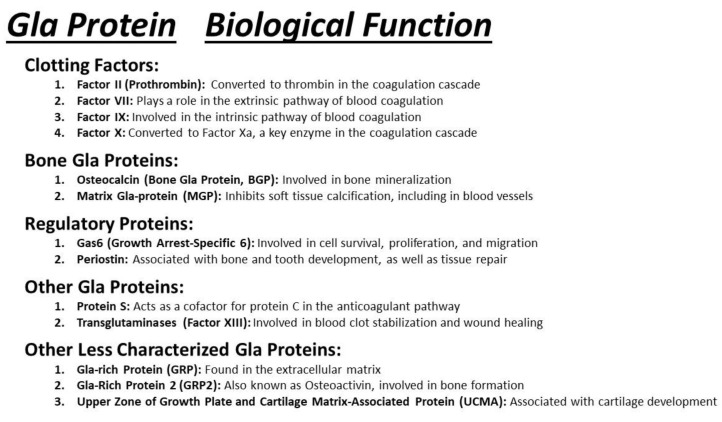
The scheme illustrates the specific Gla protein name and the categories of proteins to include clotting factors, bone proteins, regulatory proteins, other Gla proteins, as well as less well-characterized Gla proteins. Their respective biological function is also listed in this scheme.

The diverse biological functions of Gla proteins are the focus of ongoing investigation [40,41,42]. Many of these proteins play crucial roles in blood coagulation and calcium regulation within the bone and vasculature (Figure 3). Examples of extensively studied hepatic Gla proteins include Factor II (prothrombin), Factor VII, Factor IX, Factor X, Protein S, and Protein C. The liver is the main site for the synthesis of these proteins, contributing to the precise spatiotemporal control of the blood clotting cascade [32,43,44,45]. Specifically, the gamma-carboxylation ensures effective assembly and disassembly of the tenase complex by facilitating calcium and phospholipid binding. The tenase complex, in turn, plays a crucial role in activating Factor X into its active form, Factor Xa, leading to the subsequent formation of the prothrombinase complex and the conversion of prothrombin to thrombin. 

## 4. Key Forms and Sources of Vitamin K

**Figure 4:**
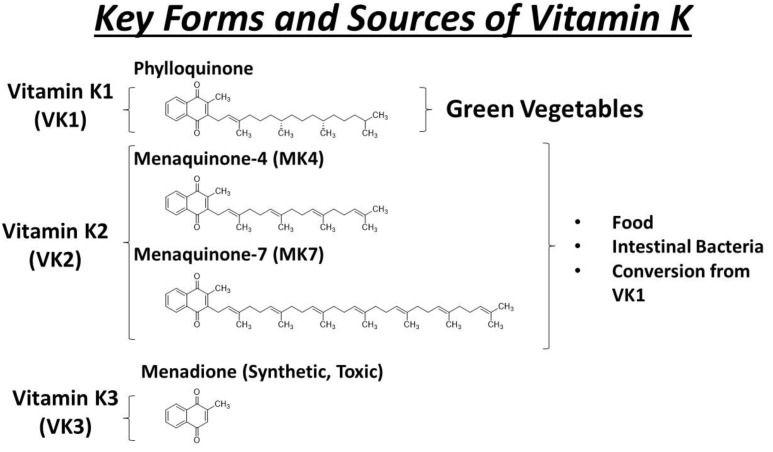
The scheme illustrates the structure, key forms, and sources of vitamin K. Vitamin K1 (phylloquinone) is primarily found in green leafy vegetables like kale, spinach, broccoli, and Brussels sprouts. On the other hand, vitamin K2 (menaquinones), which includes specific forms like menaquinone-4 (MK-4) and menaquinone-7 (MK-7), is present in animal products such as meat, eggs, and dairy, as well as fermented foods like natto—a traditional Japanese dish made from fermented soybeans known for its unique taste, slimy texture, and strong aroma. Gut bacteria play a role in converting K1 into K2 through menaquinone biosynthesis, a process that involves modifying the side chain of the vitamin K molecule. This conversion results in various forms of vitamin K2, including MK-4. MK-4 is not only produced by gut bacteria but can also be found in certain animal products, particularly in meats like chicken and pork, organ meats, and dairy products such as eggs.

Vitamin K exists in several forms, with the most prominent being K1 (phylloquinone) and K2 (menaquinone). Vitamin K1 is predominantly found in green leafy vegetables such as spinach, kale, and broccoli. It serves as a primary dietary source of vitamin K for many individuals. On the other hand, vitamin K2 encompasses various subtypes (MK-4 to MK-13), primarily derived from animal products and fermented foods. Animal sources include meat, eggs, and dairy, while fermented foods like natto (fermented soybeans) are rich in certain forms of vitamin K2, such as MK-7 (Figure 4). These different forms and sources collectively contribute to maintaining adequate levels of vitamin K in the body, supporting essential functions such as blood clotting and bone metabolism. 

The different forms of vitamin K play distinct roles in supporting various physiological processes throughout the body. Vitamin K1 (phylloquinone) is primarily involved in the hepatic synthesis of clotting factors and proteins in the liver [46]. On the other hand, extrahepatic tissues such as bone and blood vessels utilize vitamin K2 as a cofactor for the synthesis of Gla proteins, particularly menaquinone-4 (MK-4). Vitamin K2 actually comprises a group of chemical compounds with a consistent structure, featuring a naphthoquinone ring and an isoprenoid side chain of variable lengths [46]. The different compounds are denoted as MK_n (MK-2 to MK-14), where “n” signifies the number of remaining chains in the isoprenoid unit. Examples of extrahepatic Gla proteins include osteocalcin (involved in bone metabolism) and matrix Gla-protein (MGP, involved in the regulation of vascular calcification) [40,41,47]. MK-4 is crucial for the gamma-carboxylation of these proteins, allowing them to bind calcium ions and perform their respective functions in tissues outside the liver [44].

Vitamin K3, also known as menadione, is a synthetic form of vitamin K. While it was previously used as a supplement to address vitamin K deficiencies, its use has become limited due to concerns about potential toxicity [30,48,49,50]. High doses of vitamin K3 can lead to adverse effects, including liver damage and hemolytic anemia [51,52,53]. As a result, more bioavailable and less toxic forms of vitamin K, such as K1 and K2, are generally preferred for supplementation and medical interventions.

Gut bacteria also contribute to vitamin K synthesis, providing an additional source for human nutrition [54,55]. The specific bacteria that produce menaquinones in the human gut contribute to the overall vitamin K status of the individual [56]. It is important to note that the extent and efficiency of vitamin K2 synthesis in the gut can vary among individuals. The distinction between vitamin K1 and vitamin K2 is particularly relevant when considering the synthesis of clotting factors in the liver (vitamin K1) versus the gamma-carboxylation of proteins in extrahepatic tissues like bone (vitamin K2) [46]. In either case, vitamin K is crucial for blood clotting and bone health, likely through multiple molecular mechanisms.

Vitamin K is found throughout the body, including the liver, brain, heart, pancreas, and bone. It is extensively metabolized in the liver and excreted primarily in the urine (20%) and bile (40%) [57]. While both forms of vitamin K1 and K2 are involved in blood coagulation and bone metabolism, vitamin K2 has been suggested to have additional roles in cardiovascular health and possibly other physiological processes [28,29,58]. The absorption of vitamin K2 occurs in the small intestine, like vitamin K1, through passive diffusion and possibly active transport mechanisms. Factors such as dietary fat content and bile acids may influence the absorption of vitamin K2, like other fat-soluble vitamins [59]. Once absorbed, vitamin K2 is transported in the bloodstream primarily bound to lipoproteins, particularly low-density lipoproteins (LDLs) and very low-density lipoproteins (VLDLs). Vitamin K2 is distributed to various tissues, including the liver, bones, and other tissues where it is utilized for its biological functions. Like vitamin K1, vitamin K2 undergoes hepatic metabolism primarily in the liver. It is metabolized by hepatic enzymes, including cytochrome P450 enzymes, to its active form, which is involved in the gamma-carboxylation of vitamin K-dependent proteins. Like vitamin K1, the excretion of vitamin K2 and its metabolites occurs via bile into the feces. Some small amounts of vitamin K2 and its metabolites may also be excreted in urine.

In the past, indirect assessment of vitamin K serum levels relied on measurements of undercarboxylated Gla proteins. It is critical that the direct assessment of serum vitamin K levels has been achieved using liquid chromatography–tandem mass spectrometry detection [60]. Studies by Fusaro et al. point to a key role for vitamin K2 in bone metabolism and vascular calcification, and this is especially important during kidney disease and in the aging population [58,60,61]. The authors suggest that the general population could be studied for vitamin K deficiency as a cause of vertebral fracture and vascular calcification. Their studies suggests that the vitamin K system may be important for preserving bone mass and avoiding vascular calcification, particularly in hemodialysis patients. An excellent review of the protective role of vitamin K in bone and vascular health is presented by these authors [58]. Their studies further highlight the likely role of vitamin K in bone and vascular health.

## 5. MK-4 and PXR

Menaquinone-4 (MK-4), recognized for its four isoprenoid units, is a short-chain menaquinone found in certain animal-based foods and synthesized in the body from vitamin K1. MK-4, often used in dietary supplements, is the most well-known form of vitamin K2 [62]. In the context of gut, liver, and bone health, the MK-4 activation of PXR helps maintain enterohepatic homeostasis, reduce inflammation, and protect against oxidative stress and bone loss [30,41,45,63,64,65]. However, prolonged PXR activation can also contribute to various diseases, including inflammatory bowel disease and cancer. Laboratory observations suggest that PXR activation by MK-4 may reduce inflammation and oxidative stress in gut epithelial cells, promoting intestinal integrity and functionality [20,66,67,68,69,70,71]. Understanding the pivotal role of vitamin K2-mediated PXR activation is crucial for health and disease management, opening avenues for innovative therapeutic strategies. The influence of PXR on inflammation control and the reinforcement of gut barrier function holds clinical significance. These insights offer a foundation for potential interventions and therapies in gastrointestinal disorders, inflammatory bowel disease, and other conditions where maintaining intestinal homeostasis is critical [72]. Further research is needed to fully understand the clinical implications of PXR activation by vitamin K2 in the intestine, shedding light on the intricate interplay between molecular mechanisms and clinical outcomes, providing promising avenues for managing gut-related diseases.

## 6. Local Biosynthesis of MK-4

The UbiA Prenyltransferase Domain Containing 1 (UBIAD1) is a gene that codes for the protein UBIAD1, also known as TERE1 (transitional epithelial response protein 1) or MenA (menaquinone-specific isochorismate synthase [73,74]. The UBIAD1 protein localizes inside cells to mitochondria and is indeed associated with the local conversion of vitamin K1 (phylloquinone) to MK-4 (menaquinone-4), a form of vitamin K, as well as the production of ubiquinone (aka; coenzyme Q-10; CoQ10). The conversion of vitamin K1 to MK-4 in tissues other than liver is increasingly recognized as an important part of the vitamin K metabolic pathway. This conversion is very likely important for various biological functions, including the regulation of blood clotting and bone metabolism. MK-4 has been suggested to have roles beyond those of other forms of vitamin K, and its localized synthesis in tissues by UBIAD1 may contribute to its tissue-specific functions.

**Figure 5:**
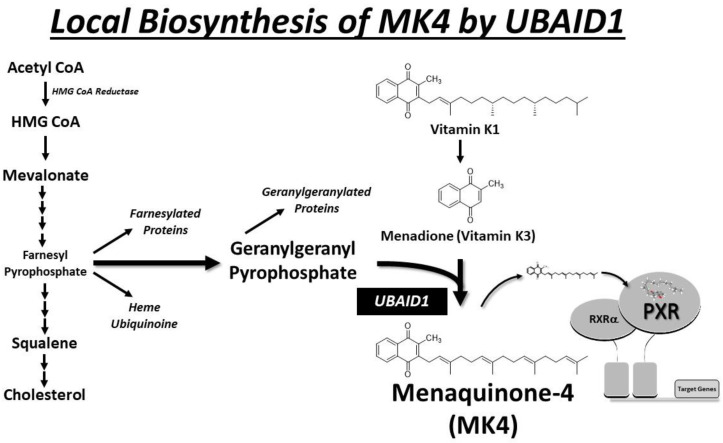
The scheme illustrates the likely pathway of local biosynthesis of MK-4 in extrahepatic tissues by UBIAD1. MK-4 stands out as the primary form of vitamin K in vertebrate animals.

It is noteworthy that the precise functions and implications of UBIAD1 and the conversion of vitamin K1 to MK-4 in different tissues are areas of ongoing research, and numerous labs continue to explore the various roles of vitamin K in health and disease. MK-4 is prominently present in various tissues such as the liver, kidney, adipose tissue, reproductive organs, bone, and pancreas. UBIAD1, a gene coding for the protein UBIAD1, is enriched in these tissues and is responsible for the local conversion of vitamin K1 to MK-4 (Figure 5). The relationship between UBIAD1, vitamin K metabolism, PXR activation, and specific health conditions is an area of ongoing research. While the exact significance of MK-4 accumulation in various non-hepatic organs is not fully understood, it is plausible that localized reservoirs may contribute to the synthesis of γ-carboxylated proteins or manifest alternative biological effects through PXR activation in these tissues. Beyond PXR activation, additional non-canonical molecular mechanisms postulated for vitamin K biological function include the activation of PKA [75], interaction with 17β-hydroxysteroid dehydrogenase [76], and interaction with Bcl-2 antagonist killer 1 (Bak) as a molecular target of VK2-induced apoptosis [77]. The body produces MK-4 through the conversion of vitamin K1, particularly in the testes, pancreas, and arterial walls [41]. Notably, this conversion is not reliant on gut bacteria, as evidenced by its occurrence in germ-free rats and when vitamin K1 is administered parenterally in rats [78,79,80,81]. Tissues that accumulate substantial MK-4 also demonstrate the ability to convert up to 90% of available K1 into MK-4 [82]. The biosynthetic enzyme UBIAD1 plays a crucial role in MK-4 production from K vitamins (PK and MKs) and the formation of cellular MK-4 pools. An unidentified enzyme cleaves the side chain of K vitamins to form menadione, which is then prenylated by UBIAD1 using the isoprenoid geranylgeranyl pyrophosphate (GGpp) to produce MK-4. UBIAD1’s translocation from the endoplasmic reticulum (ER) to the Golgi is influenced by high sterol synthesis, thereby regulating sterol synthesis and maintaining isoprenoid synthesis. MK-4, in turn, binds and activates the transcription factor PXR, leading to the upregulation of gene expression, including those involved in cholesterol catabolism and efflux. This process likely contributes to reducing the cancer phenotype.

## 7. Vitamin K2, PXR Activation, and Inflammatory Bowel Disease

It is interesting to note that vitamin D and vitamin K deficiencies are thought to be associated with a heightened inflammatory state, and moreover, micronutrient deficiencies likely occur in most IBD patients, especially with Crohn’s disease [83]. Experimental evidence suggests that PXR activation by vitamin K2 can potentially alleviate the symptoms of IBD by mitigating inflammation, maintaining intestinal homeostasis, and promoting the detoxification of harmful compounds [84,85,86]. Clinical studies and experimental evidence suggest that PXR activation by vitamin K2 can potentially alleviate the symptoms of IBD by mitigating inflammation, maintaining intestinal homeostasis, and promoting the detoxification of harmful compounds [85,86,87]. Specifically, PXR activation by a gut-specific PXR agonist rifaximin regulates the expression of genes responsible for the metabolism of inflammatory mediators and, in doing so, helps to quell the excessive immune responses that underlie IBD pathogenesis [67,68,88,89,90,91]. Additionally, PXR activation reinforces the gut barrier, reducing the permeability of the intestinal mucosa and preventing the translocation of noxious substances from the gut into systemic circulation [92,93,94,95]. These combined effects of PXR activation may hold the potential to ameliorate the chronic inflammation and tissue damage characteristic of IBD, offering hope for novel therapeutic strategies in the management of these debilitating conditions. 

## 8. Vitamin K2, PXR Activation, and Cancer

The relationship between PXR activation and cancer represents a compelling area of recent investigation. PXR, a critical regulator of xenobiotic metabolism, has emerged as a key player in this context [96]. In general, in cancer, the acquisition of drug resistance, which is commonly associated with treatment failures in cancer, may be lessened by combination therapies with different mechanisms of action, to include the vitamin K-mediated activation of PXR [32]. Clinical studies and experimental evidence indicate that the activation of PXR by specific agonists, such as vitamin K2 and others, can influence the progression of hepatocellular and colorectal cancer [87,97,98,99,100,101,102]. By targeting PXR with vitamin K2 in combination anti-cancer therapy, we may be able to modulate the response to chemotherapy and improve therapeutic outcomes for cancer patients. 

The intricate interplay between PXR and liver and colorectal cancer highlights the need for further research into this relationship, offering new insights and therapeutic possibilities in the ongoing battle against this devastating disease. Indeed, a recent review suggests a role for PXR in regulating chemotherapeutic resistance in breast cancer [22]. The authors postulate that increased expression of PXR in cancerous breast tissue indicates a potential connection between irregular cell division and the protection of cancer cells against foreign substances. Additionally, PXR has a positive impact on the cell cycle [9], possibly promoting the uncontrolled growth of certain tumor cell types. PXR activation is crucial in controlling apoptosis and contributing to acquired resistance to chemotherapy. Moreover, high levels of PXR expression are correlated with poor prognosis in invasive breast carcinoma [71]. The suppressive role of PXR in regulating inflammatory mediators, combined with genetic polymorphisms in the PXR gene sequence, may make individuals more susceptible to developing breast cancer. Additional research is needed to elucidate the mechanisms of natural and synthetic vitamin K compounds and their possible significance in cancer prevention and treatment.

PXR activation has been implicated in both promoting and suppressing cancer, depending on the context. In some cases, PXR activation can induce the expression of enzymes involved in the detoxification of potential carcinogens, thus potentially reducing cancer risk. However, PXR activation can also affect cell proliferation, apoptosis, and inflammation pathways, which may either promote or inhibit cancer development depending on the specific circumstances. The relationship between vitamin K-mediated activation of PXR and its impact on cancer initiation and progression in patients with irritable bowel syndrome (IBS) is complex and not fully understood. The effect of vitamin K-induced PXR activation on cancer initiation and progression in patients with IBS is uncertain and likely multifaceted. It could potentially have both protective and harmful effects depending on various factors such as the specific type of cancer, genetic predispositions, concurrent medications, and overall health status. While vitamin K-induced PXR activation may have implications for cancer initiation and progression in patients with IBS, the precise nature of these effects is complex and requires further research for a comprehensive understanding.

## 9. PXR Activation, Autophagy, and Bone Health

The relationship between PXR activation and autophagy is complex and appears context dependent. PXR activation has been reported to both enhance and inhibit autophagy in different cellular contexts [103]. Some studies suggest that PXR activation can promote autophagy. For example, in certain liver cells, PXR activation was associated with increased autophagic activity [104], which is considered a cellular process that helps in the removal of damaged organelles and proteins [32]. On the other hand, in other cellular environments or conditions, PXR activation has been linked to the inhibition of autophagy [105,106,107,108,109]. PXR activation has been associated with the inhibition of autophagy in hepatocytes, primarily through the modulation of the mTOR signaling pathway and potentially other mechanisms [105]. Osteoclasts are cells responsible for bone resorption, and excessive osteoclast activity can lead to bone loss. Emerging evidence suggests that PXR activation may influence osteoclast differentiation and activity [110,111]. Autophagy has been implicated in the regulation of osteoclast function, suggesting a potential link between PXR-mediated autophagy and bone resorption. While direct evidence linking PXR activation to autophagy regulation in bone is limited, its effects on bone metabolism and cellular function suggest potential indirect influences on autophagy pathways. The dual role of PXR in autophagy regulation might be influenced by various factors, including cell type, tissue context, and the specific stimuli present. The exact effect of PXR activation on autophagy in bone cells is likely context dependent and may vary based on the specific cellular environment, the presence of other signaling pathways, and the nature of the stressors or stimuli involved. PXR likely modulates bone autophagy through multiple indirect mechanisms to include the regulation of osteoblast and osteoclast function, the inhibition of osteoclast differentiation and activity by downregulating RANKL expression and modulating NF-κB signaling, and the interaction with vitamin D metabolism [112,113,114]. The complexity of these interactions highlights the need for further research to fully understand the mechanisms and implications of PXR activation in autophagy regulation. 

The interface between PXR, vitamin K, and their collective impact on bone health is another area of recent interest. MK-4 has diverse biological functions, including its role in regulating gene expression through the activation of PXR to support bone health. Laboratory investigations reveal that PXR activation by vitamin K2 in cultured bone cells, as well as in wild type mice but not in pxr nullizygous mice, induces the expression of key bone marker proteins [63,65,75]. Azuma et al. have characterized several genes that are induced by MK-4 in an PXR-dependent manner, such as tsukushi (Tsk), matrilin-2 (Matn2), and CD14 [24,110]. Tsk produces a protein with a collagen-accumulating impact, Matn2 is a protein similar to collagen in the extracellular matrix, and CD14 regulates the differentiation of B cells, influencing osteoblastogenesis and osteoclastogenesis. Additionally, 4-month-old female PXR-deficient mice exhibited lower bone mineral density in the femoral bone, with experiments revealing a fragile trabecular bone structure and bone loss in these mice [110]. Importantly, the induction of these genes was not hindered by warfarin treatment, suggesting a GGCX-independent mechanism for vitamin K in bone. There are also significant protein kinase A-mediated effects of vitamin K2 that do not require PXR [75]. Thus, there are likely multiple mechanisms by which vitamin K increases bone health to include, in part, the apparent activation of PXR in osteoblasts. In summary, PXR plays a role in promoting bone formation and inhibiting bone resorption, indicating its potential as a crucial regulator of bone homeostasis.

## 10. Therapeutic Implications of PXR Activation by Vitamin K

Ongoing research in the field of PXR activation by vitamin K and its implications for therapeutic intervention affecting human health is an area of considerable interest and promise. Current structural investigations are delving deeper into the precise mechanisms through which PXR interacts with agonists such as vitamin K2 and the intricate tissue-specific pathways it regulates [115]. Researchers are also exploring the potential development of PXR-targeted therapeutics for a range of conditions, from gut and liver disorders to cancer and inflammatory diseases. Future directions in this field could include the identification of novel PXR agonists or antagonists with enhanced specificity and effectiveness [94,116]. Moreover, understanding the genetic and environmental factors that influence PXR activity may pave the way for more personalized and precise medical interventions [117,118,119,120]. The extent to which vitamin K deficiency correlates with interindividual variability in drug metabolism is an open question that deserves investigation. The burgeoning insights into the role of PXR and its impact on clinical health outcomes are likely to catalyze further research, offering new strategies for disease prevention, management, and treatment. As ongoing studies continue to unravel the complexities of PXR activation, the prospects for innovative healthcare interventions are poised to flourish, providing new avenues for improving human health and well-being.

## 11. Conclusions

In conclusion, the exploration of PXR activation by vitamin K2 has unveiled a complex network of molecular mechanisms that transcend its conventional roles, offering new insights into PXR involvement in clinical health outcomes. This review has further underscored the multifaceted functions of PXR and its potential to impact human health, with far-reaching consequences for diseases such as inflammatory bowel disease, cancer, osteoporosis, and various other conditions. By regulating xenobiotic metabolism, inflammation, autophagy, and oxidative stress, PXR activation by vitamin K presents a valuable therapeutic target for personalized medicine, allowing for tailored interventions that consider individual patient needs. Exploring the precise molecular mechanisms responsible for conferring tissue-specific activity to the vitamin K-mediated activation of the PXR is likely to be an emerging focus of research in the near future.
